# The specifics of integrating distance learning technologies with traditional classroom instruction: How to design educational curricula in modern education?

**DOI:** 10.1016/j.heliyon.2024.e38740

**Published:** 2024-09-30

**Authors:** Natalia Zakharova, Svetlana Frumina, Liudmila Lobuteva, Suad Alwaely

**Affiliations:** a Department of Chemistry, Sechenov First Moscow State Medical University, Moscow, Russia; b Department of Global Financial Markets and Fintech, Plekhanov Russian University of Economics, Moscow, Russia; c Department of Public Finance, Faculty of Finance, Financial University under the Government of the Russian Federation, Russia; d Department of Organization and Economics of Pharmacy, Sechenov First Moscow State Medical University, Moscow, Russia; e Department in Arabic Language Curricula and Islamic Education, Al Ain University, Abu Dhabi, United Arab Emirates; f Department Curriculum and teaching, Hashemite University, Zarqa, Jordan

**Keywords:** Blended learning, Distance learning, Educational programs, Educational technologies

## Abstract

The active and widespread transition to distance learning modalities was prompted by the COVID-19 pandemic and is currently considered a prospective paradigm for the development of the education system. This shift necessitates the creation of corresponding curricula and programs capable of fostering student competencies through online and blended learning. The purpose of this research is to examine the specifics and formulate an algorithm for the development of educational programs incorporating distance and blended learning technologies. Analytical, sociological (utilizing semi-structured surveys administered to 96 educators and 128 students on the subject of distance and blended learning and its methodological support), modeling, and mathematical-statistical methods (Student's t-test and analysis of variance ANOVA) were employed in this study. The survey confirmed the hypothesis of differences in attitudes toward distance learning among students and educators. Educational needs and challenges were identified, leading to the development of an algorithm for designing educational programs integrating distance and blended learning technologies. An algorithm has been devised for the development of educational programs based on the integration of distance and blended learning technologies. The study underscores the importance of continuity across educational levels and the early cultivation of distance learning skills. *Prospective research* endeavors are intended to be directed toward the examination of the organization of distance and blended learning for students with special educational needs.

## 1 Introduction

The primary objective of contemporary educational programs is to ensure the quality of education [1]. However, a range of educational challenges exists, among which the following prevail: irrational utilization of resources; misalignment of programs and teaching strategies with the contemporary requirements and needs of educational participants; absence of modeling of student behavior; lack of quality control; and insufficient attention to compliance with moral-ethical norms [1]. Additionally, there is a correlation between the emotional intelligence of the teacher and the student, impacting the effectiveness of education [2].

The COVID-19 pandemic has had a significant impact on the education system, leading to significant reforms and restructuring, the implementation of which was accompanied by a number of problems and prejudices. Distance education is considered as the primary form of learning in the 21st century, during the complex and challenging transition from the traditional campus-based teaching concept to the new format of a digital educational environment [3]. Nevertheless, the new system of distance online learning quickly gained support among students and educators and is currently considered one of the preferred directions for the development of education [4]. The transition to distance learning through the utilization of educational platforms such as Moodle implies the creation of corresponding curricula capable of fostering the development of reflective competencies in students during online learning [5]. Online learning makes it possible to ensure the openness of educational resources, which contributes to the creation of an accessible, equal, democratic and high-quality global educational space [6].

The digitization of education poses a challenge, necessitating the search for the most effective solutions that align with the actual needs of all participants in the educational process [7,8]. The process of distance learning is characterized by complexity, associated with the imperative of ensuring the participation of all stakeholders [9]. The distance learning environment is dynamic, flexible and comfortable, since it is not bound by space-time restrictions, while simultaneously supporting and improving existing traditional training programs, increasing students' motivation to learn, their understanding of educational materials and overall satisfaction with the educational process [10,11].

However, some authors [12], conversely, highlight a crisis in e-learning. They associate this crisis with a series of issues, including the complexity of technical infrastructure, the organization of enhancing the digital competence of teachers, the need to explore and employ non-standard solutions, the search for ways to improve the efficiency of learning using electronic technologies, the adaptation and enhancement of proven applications and programs, students' readiness for independent work, their possession of necessary skills in utilizing educational platforms and programs, parents' lack of understanding of the essence of educational innovations, and the modernization of the workspaces of teachers and students by technological progress in organizing the learning process [12]. At the same time, with appropriate pedagogical approaches, online learning fosters greater student engagement, and independence, and enhances the interaction among participants in the educational process [13].

***The scientific novelty*** of the present research lies in the theoretical substantiation of the peculiarities involved in developing educational programs using distance and blended learning technologies, representing a significant innovative contribution to the theory of contemporary pedagogical science.

***The practical significance*** of the research results resides in the formulation of a scientifically grounded algorithm for the creation of educational programs incorporating distance and blended learning technologies.

## 2 Literature review

### 2.1 Characteristics of developing modern educational programs

The flexibility of educational programs is particularly crucial when introducing innovative teaching methods, as attempts to reconcile old programs with new educational technologies often fail to provide adequate feedback and hinder the objective assessment of learning outcomes. This, in turn, diminishes student engagement and satisfaction with the learning process, especially concerning the development of practical application skills [14]. However, contemporary educational trends highlight the necessity of ensuring flexibility in the curriculum without excessively complicating it [15].

The dynamic process of educational reforms has led to a situation that some researchers [16] are inclined to regard as a crisis in educational programs. These programs largely do not meet the needs of participants in the educational process, fail to ensure the necessary quality of knowledge, and lack proper control. This necessitates the development of a conceptually new model of an educational program focused on the development of students' educational and creative potential, as well as their ability for independent critical thinking [17,18]. The determining factor facilitating the integration of innovative technologies into the educational process is the educators, as student engagement in the learning process is contingent on their efforts [19].

The structure of contemporary educational programs involves a blend of didactic traditions with global innovative educational trends associated with the digitization of learning and the development of its distance and blended forms [20,21]. However, there exists a challenge in the practical implementation of these didactic theories [22].

### 2.2 The impact of the consequences of the COVID-19 pandemic on the educational paradigm

The active and widespread transition to distance learning occurred during the COVID-19 pandemic, demonstrating both the possibilities and limitations of online education [23]. In the process of shifting to distance learning during the COVID-19 pandemic, unique experiences were gained, allowing the identification of key strategies and principles of online learning. This facilitated the provision of informational assistance and support to participants in the educational process, increased student engagement and participation, and enhanced the capabilities of online learning platforms [24].

The high technological nature of distance online learning showcased its advantage in providing practically unlimited access to educational resources, utilizing virtual environments and augmented reality for learning purposes. This positions it as a crucial component within the contemporary education system and designates online learning as a priority in global educational policies [[25], [26], [27]]. However, the question remains regarding the extent to which the advancements in distance learning during the pandemic will be utilized in the long term after its conclusion [13].

The introduction of online classes and the ability to conduct lessons and assess learners using proctoring technology allowed the continuation of the educational process during the restrictions associated with the COVID-19 pandemic. This proved to be a novel experience for the majority of participants in the educational process worldwide and led to radical changes in the perception of the widespread transition to distance learning. However, it brought to the forefront the issue of aligning the information and communication technology skills of both students and educators with those necessary for ensuring quality and sustainable online learning [28]. Furthermore, the COVID-19 pandemic prompted the realization of the need to adapt curricula and programs to the characteristics and requirements of distance online education [29].

### 2.3 The transition from traditional to distance and blended learning: a direction in the development of modern education

Distance education is associated with various expectations, stereotypes, and prejudices. However, it is just one of many instructional modalities, not identical to traditional methods, and does not directly impact the quality and outcomes of education [30]. The development of the education system based on the distance online model aims to meet the dynamic educational needs of students in the contemporary high-tech era, providing the opportunity to deliver maximum information to students within a limited period [31]. Gamification has proven to be an effective tool in distance education, facilitating collaborative student learning in a digital format [[32], [33], [34]]. A modern direction in online education involves refining the capabilities of the Metaverse platform [35].

At the same time, the distance learning format is not without its challenges, such as the complexity of effectively engaging students in the learning process and the alteration of communication dynamics among participants in the educational process in the new learning environment [29,36]. Additionally, the development of distance education requires overcoming various technological and organizational issues, including ensuring ubiquitous fast access to quality internet and cloud technologies [26].

Modern realities demand not only new approaches to education but also the development of new standards for it [37]. The implementation of distance learning technologies is a comprehensive dynamic process that contributes to the activation of independent cognitive activities of students [38]. An essential task in organizing distance learning is creating a high-quality and user-friendly public educational online platform capable of meeting the needs of all participants in the educational process at various levels (school, college, university) [39]. This requires the improvement of methodological support for the educational process and the development of modern educational programs considering distance and blended learning formats.

### 2.4 Problem statement

An analytical review of scientific literature allowed for the assessment of distance learning as a promising form of education, the identification of the advantages and disadvantages of online learning, and the delineation of current issues requiring a scientific approach to their resolution. It is noteworthy that while distance learning has been applied for a long time, its widespread implementation occurred in connection with the COVID-19 pandemic. Initially, the transition to distance learning posed a series of problems related to both objective factors, such as Internet accessibility and access to resources, and subjective issues like insufficient qualifications of teachers and students' lack of readiness. However, the evident advantages of distance learning soon made it preferable compared to traditional classroom sessions.

Among the primary advantages of distance learning, flexibility, and accessibility stand out. This allows all participants in the educational process to freely access any necessary informational educational resources and organize a convenient schedule of classes, ensuring individualization and interaction. It is also believed that distance learning contributes to the increase in student motivation. However, scientific literature sources do not reveal compelling data on significant, statistically significant differences in the level of student motivation depending on the form of education [40].

Thus, the COVID-19 pandemic has contributed to the transformation of the educational paradigm by shifting the focus to distance learning. However, a significant barrier hindering the development of distance education technologies has been outdated curricula geared towards traditional classroom instruction, neglecting innovative technological changes in education. The implementation of the practical component of the educational process is particularly challenging in organizing distance learning, necessitating a qualitatively new algorithm for creating educational programs, with a mandatory requirement for technological support.

***Research Objective:*** To investigate the characteristics and develop an algorithm for creating educational programs incorporating distance and blended learning technologies.

The realization of this objective involved addressing a series of tasks aimed at finding answers to the following research questions.

Q1 How relevant is the issue of integrating distance learning technologies into the education system in the contemporary stage? What are the advantages and disadvantages of distance learning, and what are the prospects for distance educational technologies?

Q2 What are the specificities of developing educational programs incorporating distance and blended learning technologies?

Q3 What is the algorithm for integrating distance and blended learning technologies in modern educational programs?

The research hypothesis was based on the assumption of differences in attitudes toward distance learning among students and educators, which should be taken into account in the development of educational programs.

## 3 Methods and materials

### 3.1 Research design

The study comprises several stages. In the initial phase, the relevance of the research topic was determined, and an analytical review of the scientific literature was conducted. It was demonstrated that distance learning, whose global implementation occurred extensively due to the COVID-19 pandemic, possesses a range of advantages, defining it as a prospective direction for the transformation and development of the education system worldwide. However, certain drawbacks were identified. Notably, a significant obstacle to expanding the technological capabilities of education based on the development of its distance and blended forms is the mismatch between educational programs traditionally geared toward face-to-face instruction. The research goals and objectives were defined, aiming at the theoretical justification and practical development of academic programs incorporating distance and blended learning technologies.

In the second stage of the research, an experimental plan was devised, and research methods were identified, including analytical, sociological (questionnaires), modeling, and mathematical statistics. A semi-structured questionnaire was developed for surveying students and instructors regarding distance and blended learning, aimed at formulating an algorithm for creating corresponding educational programs incorporating distance and blended educational technologies. A randomized sample of research participants was formed, and their survey was conducted.

During the third stage, the obtained survey results were statistically processed and analyzed, enabling the development of an algorithm for creating fundamentally new educational programs that integrate distance and blended learning technologies.

The fourth and concluding stage involved formulating conclusions and determining the prospects for further research.

### 3.2 Sample

The sampling method employed involved simple randomization (random selection) to recruit faculty members and students from four universities and five colleges. Initially, 250 questionnaires were distributed (100 for faculty and 150 for students). All participants completed the questionnaires electronically via Google Forms. After reviewing the questionnaires for completeness and accuracy, 96 faculty questionnaires and 128 student questionnaires were accepted. The average age of faculty respondents was 45.9 years, while the average age of student respondents was 22.4 years.

Since the questionnaires were distributed across universities, participants were randomly selected from various departments, including education, psychology, computer science, pharmacy, and finance. This approach provided a broader perspective without focusing on the specifics of individual professions. Differentiation by other characteristics, such as gender or ethnicity, was not conducted, as the primary criterion was participation in the educational process.

### 3.3 Survey

To explore the opinions of participants in the educational process regarding distance and blended learning and its methodological support, a specialized semi-structured questionnaire was devised (see Appendix A). This questionnaire consisted of a series of statements, and responses were assessed on a 5-point Likert scale (1 - strongly disagree; 2 - disagree; 3 - undecided; 4 - agree; 5 - strongly agree). This design facilitated the statistical analysis of the obtained results. Additionally, the questionnaire included several open-ended questions aimed at evaluating the individual opinions of each participant on the researched issue. When developing the questionnaire, considerations were taken from the development of similar surveys published by various authors [41,42].

The questionnaire comprised two sets of questions. The first set allowed for the assessment of the current situation regarding distance learning and the attitudes of both students and instructors toward it. The second set was focused on identifying specific features that should be taken into account when developing educational programs incorporating both distance and blended learning. Some survey questions were designed in such a way that they either duplicated each other or expressed opposing attitudes. This made it possible to obtain more accurate and objective data and to clarify the characteristics of respondents’ opinions on each aspect of the problem being studied. Each questionnaire was accompanied by a detailed set of instructions for completion. Anonymity was ensured through the implementation of specific encryption measures.

The reliability of the questionnaire is assessed using the correlation coefficient. The methodology is considered reliable, given that the obtained coefficient is −0.74. To evaluate the validity of the methodology, the concordance coefficient was selected. This coefficient yielded a value of no less than 0.66, indicating a good level of validity for the methodology.

### 3.4 Statistical processing

Student's t-test and Analysis of Variance (ANOVA) were employed for the statistical analysis of the questionnaire responses. Student's t-test was selected for comparing means across different samples due to its effectiveness in analyzing small sample sizes. This test allows for the determination of whether there is a statistically significant difference between the means of two independent groups (e.g., between faculty and students). The primary reasons for employing the *t*-test are its simplicity and effectiveness. When data are normally distributed, the *t*-test provides accurate results. ANOVA analysis of variance was used as a more modern and powerful statistical analysis tool to determine how much difference there is between the indicators being compared. ANOVA (Analysis of Variance) is appropriate when it is necessary to compare means across multiple groups (e.g., among students from different faculties). ANOVA enables the examination of interactions between various factors, which can be valuable for a more in-depth understanding of the impact of different variables on the outcomes. Accumulation, sorting, and visualization of information during the research were conducted using Microsoft Excel. Calculation procedures were executed utilizing the online calculator provided by Social Science Statistics.

## 4 Results and discussion

This article provides an innovative analysis of the attitudes of both students and instructors toward the transition to distance learning. Additionally, it proposes an algorithm for developing educational programs that incorporate both distance and blended learning approaches.

First and foremost, the teaching and learning experience utilizing distance online technologies was examined through surveys administered to instructors and students. The survey results, presented in Table 1 with average scores on a 5-point Likert scale, indicate that, for the majority of survey items, there are no significant differences between the responses of instructors and students (p > 0.05). As evident from this table, both groups of respondents highlighted the flexibility of distance learning as an advantage, emphasizing the ability to organize time appropriately, as well as providing an individualized approach to each student and ensuring accessibility and equal opportunities. However, while distance learning technologies offer almost unlimited access to online educational resources, the use of non-digitized library collections, on the contrary, decreases.

**Table 1:** The comparative survey results of instructors (A) and students (B) on the use of distance and blended learning formats.

Question Block A	GPA		Student's t-test	p	ANOVA (f-value)	p
	A	B				
1.	4.3 ± 0.3	4.2 ± 0.4	0.61	0.28	0.71	0.42
2.	4.0 ± 0.8	3.4 ± 1.2	1.31	0.11	0.76	0.22
3.	3.9 ± 0.6	3.7 ± 0.2	0.98	0.18	1.35	0.29
4.	3.6 ± 1.0	3.7 ± 0.8	−0.25	0.41	0.06	0.81
5.	3.5 ± 0.5	3.3 ± 0.7	0.73	0.24	0.53	0.49
6.	3.9 ± 0.6	2.8 ± 0.4	4.47	0.00	20.77	*0.00*
7.	4.2 ± 0.3	3.4 ± 0.6	3.69	0.00	13.62	*0.01*
8.	4.1 ± 0.5	3.9 ± 0.2	1.14	0.14	1.29	0.29
9.	4.5 ± 0.2	4.6 ± 0.3	−0.82	0.22	0.39	0.55
10.	4.2 ± 0.6	4.4 ± 0.5	−0.80	0.22	0.63	0.45
11.	4.6 ± 0.3	3.8 ± 1.0	7.54	0.00	5.77	0.04
12.	4.5 ± 0.3	4.7 ± 0.2	−1.63	0.70	2.67	0.14
13.	4.3 ± 0.6	3.2 ± 0.8	3.44	0.00	7.61	0.02
14.	2.6 ± 0.7	2.2 ± 0.9	1.10	0.15	1.21	0.30
15.	4.5 ± 0.3	4.4 ± 0.1	0.94	0.19	0.00	1.00
16.	2.6 ± 0.8	3.5 ± 0.2	−3.40	0.00	9.14	0.16
17.	2.4 ± 0.5	3.0 ± 0.1	−3.63	0.00	2.83	0.13

***Note***: The value of the mean score in the tables (GPA) represents the average rating given by the study participants for each statement, which they evaluated on a scale from 1 to 5.

The study participants rated the necessity of additional digital skills for remote learning highly (GPA of 3.9 for Group A and 2.8 for Group B), with a statistically significant difference observed (p-value = 0.00). This indicates that Group A has better adapted to the technological demands of remote learning. Participants in Group A perceive a decline in teaching quality in remote learning (GPA of 4.2), compared to Group B (GPA of 3.4). This statement also shows a significant difference (p-value = 0.00), reflecting divergent perceptions of teaching quality between the two groups.

Teachers prefer communication with students in the classroom, considering it more productive (4.6 ± 0.3 points), students are less in agreement with this statement (3.8 ± 1.0 points, p < 0.05). Teachers are significantly more conservative regarding the effectiveness of distance learning compared to in-person, compared students (p < 0.05). One of the significant challenges of implementing distance learning mentioned by teachers is the lack of necessary skills (3.9 ± 0.6), whereas students consider their technological digital competence to be sufficient (2.8 ± 0.4 points, p < 0.05).

Preferential treatment was given to traditional forms of education by 37 (38.5 %) teachers and 29 (22.6 %) surveyed students. Distance learning was favored by 23 (23.9 %) teachers and 35 (27.3 %) students. Blended learning was preferred by 36 (37.5 %) teachers and 64 (50.0 %) students. The second part of the questionnaire aimed to explore the features of the methodological support of distance learning. The obtained results are presented in Table 2.

**Table 2:** The comparative evaluation by teachers (A) and students (B) of the features of methodological support for distance and blended learning.

*Question* Block B	GPA		Student's t-test	p	ANOVA (f-value)	p
	A	B				
1.	4.6 ± 0.2	4.4 ± 0.3	1.63	0.07	2.67	0.14
2.	2.8 ± 1.6	3.0 ± 0.5	−0.14	0.45	0.14	0.72
3.	4.5 ± 0.3	4.8 ± 0.2	−2.45	*0.02*	6.00	*0.04*
4.	4.3 ± 0.4	4.5 ± 0.3	−1.22	0.13	1.48	0.26
5.	4.4 ± 0.5	4.5 ± 0.2	−0.57	0.29	0.19	0.63
6.	4.6 ± 0.2	4.8 ± 0.1	−2.53	*0.02*	6.47	*0.03*
7.	4.5 ± 0.3	4.6 ± 0.2	−0.82	0.22	0.67	0.44
8.	4.6 ± 0.3	4.5 ± 0.4	0.61	0.28	0.37	0.56
9.	2.2 ± 0.5	2.9 ± 0.1	−4.24	*0.00*	17.98	*0.00*
10.	3.5 ± 1.1	4.0 ± 0.2	−1.40	0.10	1.97	0.20
11.	4.3 ± 0.5	2.8 ± 0.4	7.47	*0.00*	52.33	*0.00*
12.	3.3 ± 0.6	3.5 ± 0.8	−0.63	0.27	0.39	0.55
13.	4.4 ± 0.3	4.6 ± 0.2	−1.63	0.07	9.29	*0.16*
14.	4.6 ± 0.4	4.5 ± 0.2	0.67	0.26	0.45	0.52
15.	3.4 ± 0.6	2.3 ± 0.2	5.37	*0.00*	32.04	*0.00*
16.	3.8 ± 0.2	3.0 ± 0.1	10.12	*0.00*	8.07	*0.02*
17.	4.2 ± 0.4	3.5 ± 0.2	4.72	*0.00*	22.27	*0.00*
18.	3.3 ± 0.2	2.6 ± 0.4	5.40	*0.00*	24.22	*0.00*

***Note***: The value of the mean score in the tables (GPA) represents the average rating given by the study participants for each statement, which they evaluated on a scale from 1 to 5.

As the survey results indicated, there was less consensus among students and teachers in assessing the features of the methodological support for distance learning. They did not consider distance learning as preferable and promising (3.4 ± 0.6 points) and believed that distance education was a necessary measure only during the crisis associated with the pandemic, after which it was advisable to return to traditional classroom learning.

The difference in perception regarding the inclusion of distance and blended learning in educational programs was statistically significant (GPA of 4.5 for Group A and 4.8 for Group B, p-value = 0.02), indicating greater support for this practice in Group B. Group A was less inclined to assess students' knowledge solely through online testing (GPA of 2.2), compared to Group B (GPA of 2.9), a statistically significant difference (p-value = 0.00). This suggests that Group A has some reservations about the objectivity of online testing. Study participants believe that practical sessions should be conducted exclusively in an offline mode (GPA of 4.3 for Group A and 2.8 for Group B, p-value = 0.00). This indicates significant support for the traditional approach to practical sessions in Group A.

Students, on the contrary, were more supportive of the transition to distance learning and the continuation of online classes after the pandemic and the establishment of a distance learning system in each educational institution. At the same time, instructors consider independent student work to be a more crucial element of the curriculum, while students themselves attach significantly less importance to this issue. Regarding the structure of educational programs, both instructors and students emphasize the need for continuity between educational levels and the early development of distance learning skills, starting from preschool and early school age. Both groups of respondents highlight the importance of methodological support for full-fledged distance learning activities, although, for students, this factor proves to be more significant.

**Fig. 1:**
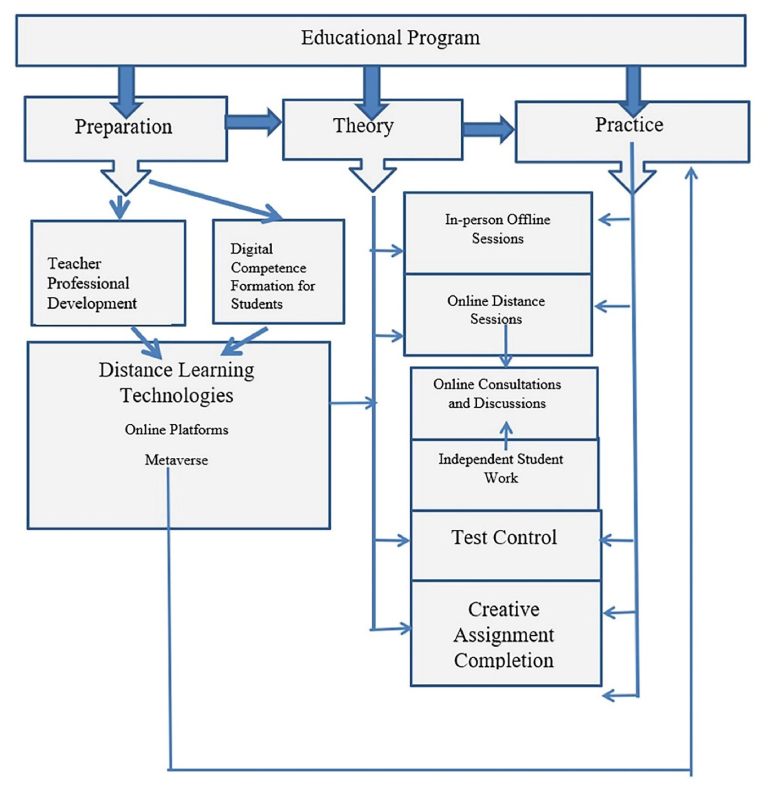
The Algorithm for developing an educational program using distance and blended learning technologies.

Based on the analysis of the conducted survey, an algorithm for developing educational programs using distance and blended learning was formulated, and visually presented in Fig. 1. The developed algorithm comprises three blocks: Preparation, Theory, and Practice. These blocks were identified as a result of analyzing the responses of teachers and students to survey questions.

The Preparation block involves the enhancement of teachers' qualifications, overcoming stereotypes and prejudices regarding digital technologies and distance learning, fostering readiness to embrace educational innovations, and developing skills in using online platforms and the possibilities of virtual and augmented reality within the context of the Metaverse.

Even though, during the survey, the majority of students assessed their digital preparedness as sufficient, it is still advisable for them to undergo preliminary training to confidently utilize the capabilities of educational online technologies and develop the ability for independent work. Moreover, the preparatory stage for students implies an early initiation not only at the college and university levels but also during preschool and school education.

Distance learning can be employed in both the theoretical and practical training of students either in isolation or in combination with traditional classroom sessions. In the case of isolated distance learning, the practical component involves leveraging the possibilities of virtual and augmented reality within the context of the Metaverse.

Furthermore, while assessing theoretical knowledge may suffice through current and final test controls, evaluating the development of practical competencies, skills, and abilities suggests undertaking a creative assignment, feasible in both offline and online formats.

An essential aspect involves providing opportunities for online consultation. This includes organizing online discussions during distance sessions and independent student work. The goal is to enhance interaction among educational participants and facilitate a comparative self-assessment of student learning.

The study aimed to explore the attitudes of teachers and students towards distance education and, based on this, develop an algorithm for creating educational programs to enhance the methodological support for distance and blended learning.

As demonstrated by the experience of the COVID-19 pandemic, information and communication technologies and distance learning formats possess undeniable advantages, positioning them as a key foundation for the prospective development of education [39]. The rapid advancement of digital information and communication technologies has stimulated the professional growth of educators, elevated the quality of teaching and learning activities in the educational environment, and fostered creative and informal approaches to organizing the learning process [43]. However, the conducted research revealed a conservative stance among many educators regarding the adoption of innovative technologies, a lack of trust in online learning, and a perception of it as a forced temporary measure during crisis situations. Conversely, students enthusiastically embrace the transition to distance education due to its flexibility, cost-effectiveness, and accessibility. Nevertheless, they exhibit insufficient readiness for independent work, which is a crucial component of online learning.

Thus, the COVID-19 pandemic emerged as a factor contributing to the shift towards online teaching and learning, forecasting the prospective directions of higher education development, and the widespread adoption of distance and blended learning formats [44]. Researchers [45] highlight a significant positive effect of transitioning to online learning in terms of improving students' academic performance. However, they emphasize the need to address a range of pertinent issues and challenges, such as ensuring the objectivity of assessment, fostering high student motivation, enhancing their readiness for self-directed learning, students acquiring professional competencies, and mastering both theoretical knowledge and practical skills during distance learning [46]. The results of the conducted research affirm the existence of these problems, suggesting avenues for their resolution to be considered in the development of new educational programs.

The advantages of distance education platforms include their convenience and user-friendly interfaces, which allow for meeting students' actual educational needs [47]. While digital technologies offer numerous opportunities, they simultaneously pose several limitations, primarily related to the characteristics of computers and the digital environment [48]. The research revealed insufficient competency among some educators regarding digital technologies and distance learning. Additionally, there is a lack of critical self-assessment among students concerning their digital skills. Therefore, the algorithm for developing educational programs, incorporating distance and blended learning, includes a preparatory phase for participants in the educational process.

The evaluation of the effectiveness of distance education contributes to optimizing both teaching methods and approaches to learning, as well as the management of educational personnel, achieving a high level of qualifications [49,50]. For administrators of educational institutions transitioning to distance and blended learning, the task involves restructuring, improving, and implementing new digital learning programs. Researchers recommend using the technology acceptance model for their assessment, which consists of four components: perceived usefulness, perceived ease of use, behavioral intentions, and actual usage [33,51]. The professional development and qualification enhancement of educators, aimed at cultivating readiness to work in the modern educational environment utilizing distance and blended learning, should involve familiarization with contemporary teaching methods, exploring the possibilities of implementing e-learning modules, and fostering the overall cultural competence of instructors [52]. These aspects have also been taken into account in the development of the algorithm for creating educational programs incorporating distance and blended learning.

Pedagogical theorists correlate motivation for learning with the elements of surprise, interest, and teacher-initiated stimulation of students' emotional involvement [53]. The research identified challenges in fostering interaction between students and educators, the intricacies of working within academic groups in an online format, and the lack of an emotional component. Technological innovations associated with the shift to distance and blended learning necessitate programmatic and methodological transformations in educational models to enhance student engagement and facilitate their acquisition of relevant scientific and technological competencies [54]. To address this issue, the developed algorithm for creating educational programs utilizing distance and blended learning incorporates the organization of online consultations and discussions, serving as a crucial factor in ensuring student motivation, and engagement, and fostering constructive interaction among educational participants.

The possibilities of utilizing Metaverse technologies in the educational process are associated with its attributes as a multi-user network of social immersive environments, blending physical reality with digital virtuality. It facilitates multi-sensory dynamic interactions among users in real-time with digital artifacts, virtual environments, and virtual (VR), and augmented (AR) reality. This provides unlimited opportunities for creating an educational online environment [55]. Considering the insufficient awareness of some participants in the educational process regarding the potential applications of Metaverse technologies in learning and the presence of subjective barriers to their integration (mainly from the perspective of educators), this aspect has been incorporated into the preparatory phase of the developed algorithm for creating educational programs with the inclusion of distance and blended learning.

The process of reforming the education system with the expansion of distance and blended learning is taking place today at all educational levels on a global scale, considering the dynamism and unpredictability of the modern world [56]. Therefore, the development of an algorithm for creating educational programs with the inclusion of distance and blended learning is considered a crucial stage in the path of technological transformations. These transformations are aimed at increasing the level of satisfaction of all participants in the educational process with its efficiency, quality, and compliance with the current and future needs of socio-economic development at both regional and global levels [56].

### 4.1 Research limitations

Limitations of the study were associated with its online format, whereas in-person interaction with respondents might have revealed additional aspects of the investigated problem. Additionally, a semi-structured questionnaire was used, allowing for understanding each respondent's perspective through the use of open-ended questions. However, if a hybrid questionnaire were used, it could have provided more diverse information on the research topic, but such a survey would have required more time for completion and analysis. Therefore, in this pilot study, we confined ourselves to a semi-structured questionnaire, the content of which validates its relevance to the studied issue of instructional support for distance and blended learning.

### 4.2 Ethical issues

The research was conducted with the approval of the ethical committees of educational institutions whose staff and students participated in the survey. All respondents provided written informed consent to participate in the study. The anonymity of research results was ensured through the use of special encryption for the questionnaires. Throughout the study, all other norms of bioethics were strictly adhered to.

## 5 Conclusions

The study revealed that both students and faculty members valued the flexibility of distance learning, noting its advantages in time management and individualized approaches to each student. The mean scores for this aspect were 4.3 ± 0.3 for faculty and 4.2 ± 0.4 for students, indicating a similarity in the perception of distance learning flexibility. However, certain challenges were identified, such as difficulties in organizing work and interaction within academic groups (4.5 ± 0.2 for faculty and 4.6 ± 0.3 for students). Faculty members were more conservative regarding distance learning, as reflected in their higher ratings for traditional teaching (4.6 ± 0.3) compared to students' ratings (3.8 ± 1.0), indicating a significant difference in perception between the two groups (p < 0.05). Additionally, faculty members believe that distance learning diminishes the quality of teaching (4.2 ± 0.3 for faculty versus 3.4 ± 0.6 for students, p < 0.05), whereas students are less critical of this aspect.

The findings of the study hold significant practical value for the development and enhancement of educational programs. Specifically, they underscore the necessity of integrating blended learning formats into the educational process, as half of the students (50.0 %) and a substantial proportion of faculty members (37.5 %) preferred blended learning. The scientific significance of the research lies in its analysis of attitudes toward distance learning within the context of contemporary educational realities. This analysis may serve as a foundation for further research aimed at improving the methodological support for distance education, as well as for examining the impact of emerging technologies, such as virtual and augmented reality, on the educational process.

Based on the obtained data, it is recommended for future research to 1) develop educational programs that integrate distance and blended learning formats, particularly for the development of practical skills; 2) address the conservative attitudes of faculty towards distance learning by creating methodological recommendations aimed at reducing stress levels and enhancing teaching quality in remote conditions; 3) implement digital skills enhancement programs for both students and faculty to address disparities in technology proficiency. Future studies may focus on examining the effectiveness of incorporating virtual and augmented reality into the educational process for the development of students' practical skills.

## Ethics approval

The authors declare that the work is written with due consideration of ethical standards. The study was conducted in accordance with the ethical principles approved by the Ethics Committee of Sechenov First Moscow State Medical University (Protocol No 356 of February 13, 2024).

## Informed consent

Informed consent was signed by participants.

## Funding

This research received no specific grant from any funding agency in the public, commercial, or not-for-profit sectors.

## Availability of data and material

Data will be available on request.

## CRediT authorship contribution statement

**Natalia Zakharova:** Writing – review & editing, Writing – original draft, Formal analysis, Data curation, Conceptualization. **Svetlana Frumina:** Writing – review & editing, Project administration, Methodology, Investigation, Funding acquisition, Formal analysis. **Liudmila Lobuteva:** Writing – review & editing, Software, Resources, Project administration. **Suad Alwaely:** Writing – review & editing, Visualization, Validation, Supervision.

## Declaration of competing interest

The authors declare that they have no known competing financial interests or personal relationships that could have appeared to influence the work reported in this paper.

## Figures and Tables

**Table:** 

No.	Questions	Absolutely disagree	Mostly disagree	Unsure	Mostly agree	Absolutely agree
		1	2	3	4	5
1.	Distance learning is more flexible and allows better organization of time.					
2.	Distance learning is less costly than traditional learning.					
3.	Distance learning provides equal opportunities and better access to educational resources.					
4.	Distance learning is associated with difficulties in accessing library resources.					
5.	Distance learning requires significant expenses for acquiring mobile devices, and computers, and ensuring quality internet access.					
6.	Distance learning requires additional digital technological skills.					
7.	The quality of teaching decreases with distance learning.					
8.	Distance learning is more individualized.					
9.	It is challenging to organize work and interaction in academic groups during distance learning.					
10.	There is a lack of emotional components in distance learning.					
11.	Interaction between students and teachers in traditional classroom settings is more productive than in distance learning.					
12.	It is difficult to develop practical skills in distance learning.					
13.	Traditional classroom learning is more effective than distance learning.					
14.	The grading system is more objective in distance learning.					
15.	It is challenging to ensure objective control of students' progress in distance learning.					
16.	The quality of education does not depend on its form.					
17.	It is difficult to ensure inclusivity for students with special educational needs in distance learning.					
18.	Education should be: A) traditional B) distance C) blended					
19.	How did the transition to distance learning affect your studies/work?					
20.	In your opinion, what are the most promising directions for the development of education?					

**Table:** 

No.	Questions	Absolutely disagree	Mostly disagree	Unsure	Mostly agree	Absolutely agree
		1	2	3	4	5
1.	Existing educational programs do not correspond to modern technological innovations in education.					
2.	The development of distance education is independent of the content and structure of educational programs.					
3.	Educational programs should include distance and blended learning formats.					
4.	The development of distance learning skills should begin in preschool and early school age.					
5.	Educational programs should be formed considering the balance between traditional and distance learning formats.					
6.	Full-fledged distance sessions should be provided in educational programs.					
7.	Educational programs should include online consultations.					
8.	Educational programs should provide the opportunity for online discussions.					
9.	Student knowledge assessment should only be conducted through online testing.					
10.	Each educational institution should establish a distance learning system.					
11.	Practical sessions should only be conducted in offline format.					
12.	The development of virtual and augmented reality technologies in the context of the Metaverse allows for conducting practical sessions in the format of distance online learning.					
13.	Educational programs should incorporate the use of virtual and augmented reality technologies.					
14.	Educational programs should maximally incorporate the use of various digital technologies and educational platforms.					
15.	Technological advancement in education and its transition to predominantly distance form are such distant and doubtful prospects that there is no sense in making any changes to existing educational programs.					
16.	Current educational programs fully comply with regulatory requirements and can be adapted to the needs of distance learning without significant changes.					
17.	Educational programs should include more independent student work.					
18.	Distance learning was a forced measure during the pandemic, and there is no basis for continuing this practice.					
19.	My experience with the use of distance and blended learning technologies shows that …					
20.	The combination of traditional classroom and distance learning is possible only under the condition …					
